# Prevalence of Intestinal Parasitic Infestation Among Expatriate Workers

**DOI:** 10.7759/cureus.4894

**Published:** 2019-06-13

**Authors:** Azhar Hussain, Eman Z Younis, Adela H Elamami, Mehrdad Jelodar, Tulika Mishra, Gopikumar Shivaramaiah

**Affiliations:** 1 Medicine, Xavier University School of Medicine, Oranjestad, ABW; 2 Laboratory Medicine, University of Benghazi, Benghazi, LBY; 3 Internal Medicine, University of Benghazi, Benghazi, LBY; 4 Internal Medicine, Xavier University School of Medicine, Oranjestad, ABW; 5 Health Biotech, Immunology, Microbiology, Xavier University School of Medicine, Oranjestad, ABW; 6 Physiology, Xavier University School of Medicine, Oranjestad, ABW

**Keywords:** expatriate workers, intestinal parasites, cryptosporidium parvum, blastocystis hominis, giardia lamblia, entamoeba histolytica, entamoeba dispar

## Abstract

Background

Parasitic infestations of the gastrointestinal tract remain a common problem in third-world countries. Poverty, illiteracy, poor hygiene, scarcity of potable water, as well as the hot and humid tropical climate, are all contributing factors associated with intestinal parasitic infestation.

Objective

This cross-sectional study aims to evaluate the prevalence of intestinal parasitic infestation amongst expatriate workers in Benghazi City, Libya.

Patients and methods

A total of 250 stool samples (200 male and 50 female) were randomly collected between October 2017 to April 2018 from expatriate workers in Benghazi City, Libya. The samples examined were used to detect the presence of intestinal parasitic infestation while the study utilized a pre-tested structure. Cases were matched based on demographic parameters, such as age, gender, and nationality, while the history of diarrhea was recorded using direct smear microscopy for the detection of intestinal parasitic infestation.

Results

Of the 250 immigrants looking for work, 95 (38%) were found to be infested with two or more intestinal parasites. The protozoa included: Blastocystis hominis, Giardia lamblia, Entamoeba histolytica, Entamoeba dispar, and Cryptosporidium parvum (47.4%, 38.9%, 17.9%, 17.9%, and 4.2%, respectively); the non-pathogenic protozoa included the prevalence of Entamoeba coli (E. coli), which is 12.6%, and the helminth Ascaris lumbricoidesis 1.1%.

Conclusion

The prevalence of parasitic infection was relatively high (38%) and was affected by individual hygiene. Therefore, comprehensive healthcare education aimed at reducing parasitic infestation is needed.

## Introduction

Intestinal parasitic infestations are distributed throughout the world, with a high prevalence in the poor and socio-economically depressed communities in the tropics and subtropics. These infestations have a clear association with human malnutrition [1], and it is a serious public health threat globally [2]. The high frequency of intestinal parasites in the population of a region indicates low socio-economic developmental conditions, poor medical care, occupational exposure, and a low standard of hygiene [3-4]. Parasitic infections, particularly intestinal helminths, cause hundreds of thousands of avoidable deaths each year and are among the world’s common infectious diseases. Intestinal helminths are more prevalent throughout the tropics, especially among poor communities. Records show increasing trends in helminthiasis, particularly in developing nations [5]. This is a public health problem with an estimated 3.5 billion people being infected worldwide, as the majority affected are children [6-7]. Socio-economic factors, such as poor hygiene, shortage of safe water supply and sanitation facilities, and low socioeconomic status, are known to play a pivotal role in susceptibility to infection [8].

Aim of the study

This study was conducted to determine the prevalence of intestinal parasites and to identify the factors that contribute to the spread of intestinal parasites among expatriate workers in Benghazi, Libya.

## Materials and methods

Study area

Located in North Africa, along the Mediterranean Sea, Benghazi is the second largest city in Libya. It is seen neighboring Egypt, Sudan, Chad, Niger, Algeria, and Tunisia.

Study design and patients

A cross-sectional study was conducted in Benghazi city. The study included 250 stool samples (200 male and 50 female), including samples from the Anti-Illegal Immigration Agency of the Ministry of the Interior, during the period from October 2017 to April 2018. Cases were matched according to demographic parameters such as age, gender, and nationality. A history of diarrhea was recorded.

Collection of samples

A single stool sample of about 2-5 grams was obtained from each individual for the expected parasites; each sample was collected fresh in a sterile plastic vial, then properly identified, and immediately transferred to the medical laboratory department of the Institute of Benghazi. The stool samples were assessed by direct smear (± 2 mg of feces) in physiologic saline solution, direct smear in Lugol’s iodine solution, formaldehyde-ether sedimentation method, and by the modified acid-fast stain in order to identify oocysts of opportunistic coccidian intestinal parasite [9]. All the samples were then placed in 10% formalin as a fixative, and a questionnaire was distributed to the expatriate workers. The questionnaire was prepared to collect the sociodemographic and clinical data from each participant, including age, gender, and nationality.

Statistical analysis

Data entry and data analysis were done using Statistical Package for Social Science (SPSS), software version 17 (IBM Corp, Armonk, NY, US). A descriptive analysis was performed for demographic findings and categorical variables. The χ2-test was employed to find the significance or non-significance of the relationships between age, sex, and symptoms and the presence or absence of parasites. The accepted level of significance P < 0.05 was considered significant [10].

## Results

**Figure 1:**
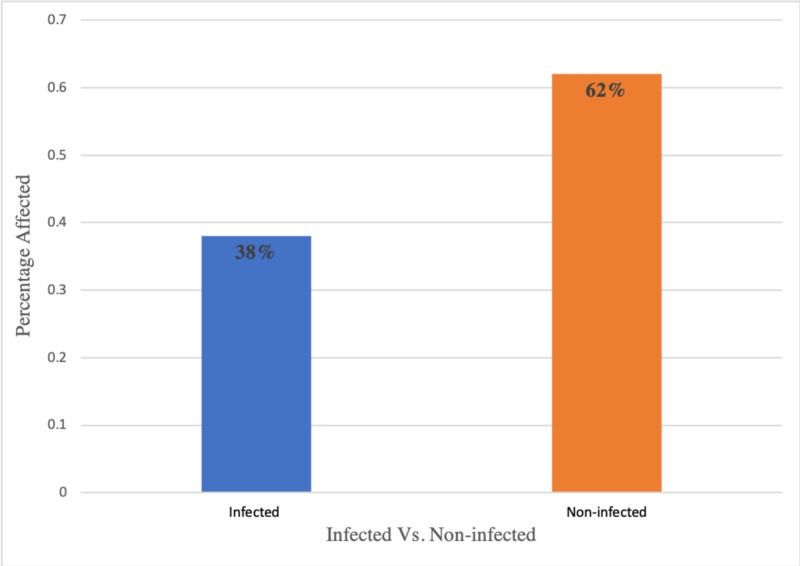
Percentage distribution of parasitic infestations amongst various populations

As illustrated in Figure 1 and Table 1, the overall prevalence of infestation with intestinal parasites among various populations was 38% (95/250) of the patient population.

**Table 1:** Overall distribution of intestinal parasitic infestations amongst various populations

Number examined	Infected	Non-infected
250	95	155

**Figure 2:**
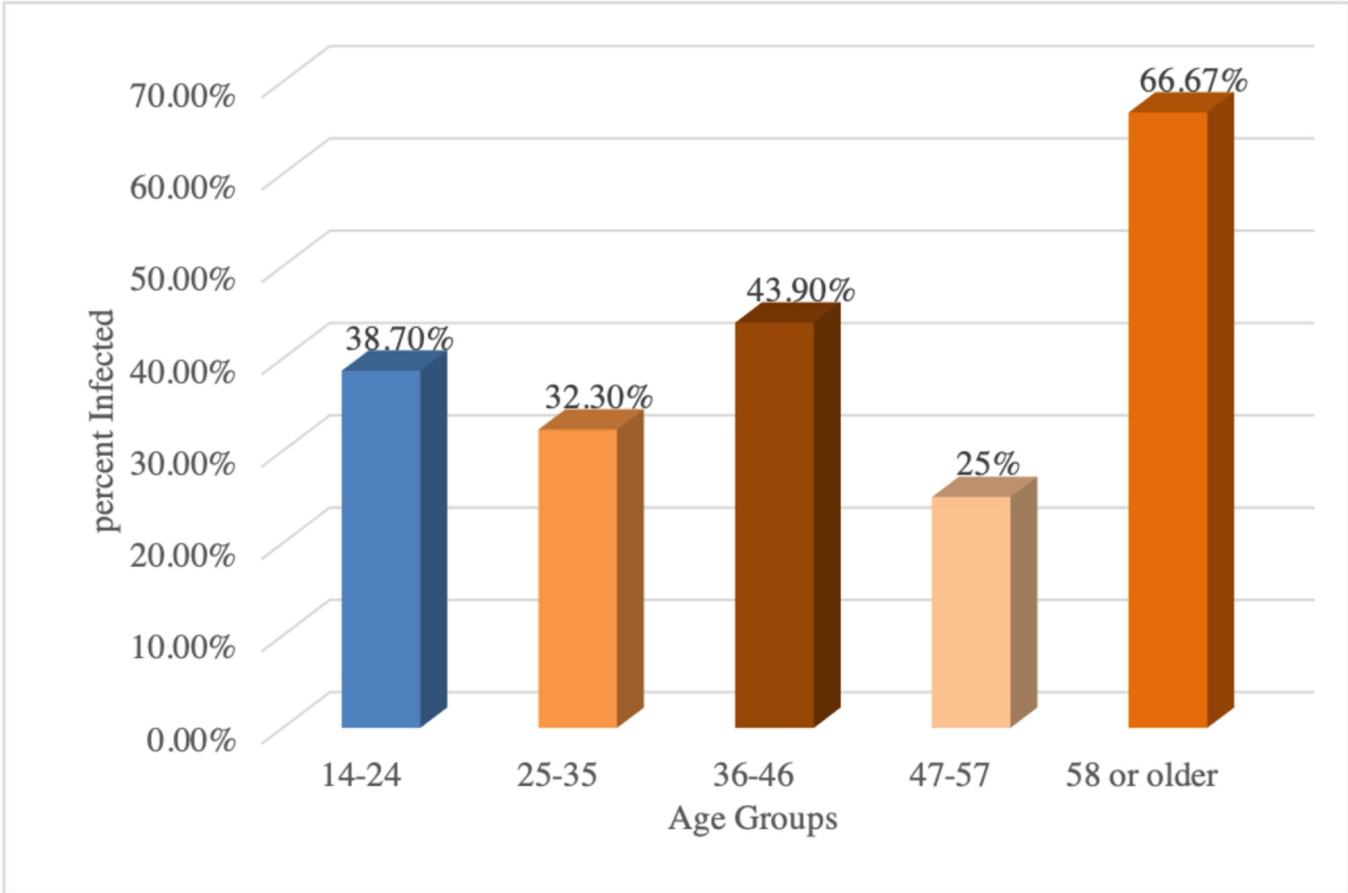
Percentage distribution of intestinal parasites amongst various age groups

These results suggested that the patients, aged 58 and older (66.7%), were the most frequently infested, followed by the age range of 36-46 years (66.7%). There were no significant differences between age group and infestation (P > 581). Our results, as shown in Table 2 and Figure 2, revealed that the prevalence rate of infestation was higher in males (85/200 (42.5%)) as compared to females 10/50 (20%) in expatriate workers.

**Table 2:** Distribution of intestinal parasites according to age group amongst various populations p= 0.581

Infection	Age					Total
	14-24	25-35	36-46	47-57	58 or older	
Positive count % within parasitic infestation	53	21	18	1	2	95
Number examined	137	65	41	4	3	250

**Figure 3:**
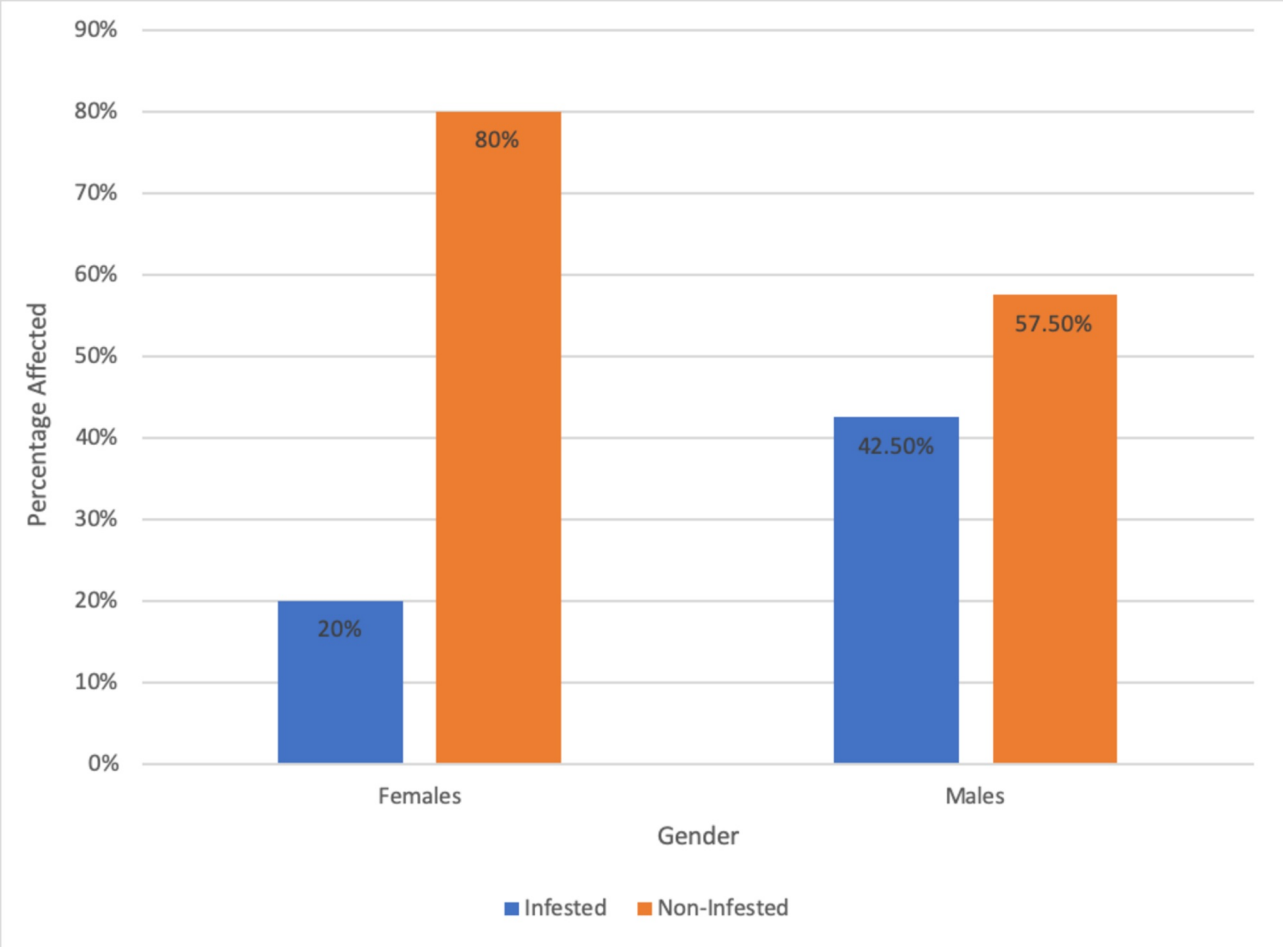
Percentage showing the relationship between the prevalence of intestinal infestation and sex amongst expatriate workers

A significant difference (P=0.000) was found between males and females. The relationship between the prevalence of intestinal parasitic infestation in expatriate workers and sex are presented in Table 3 and Figure 3.

**Table 3:** Relationship between the prevalence of intestinal parasitic infestation and sex among expatriate workers P-value = 0.000

Gender	Female	Male
Infested	10	85
Non-infested	40	115
Total	50	200

**Figure 4:**
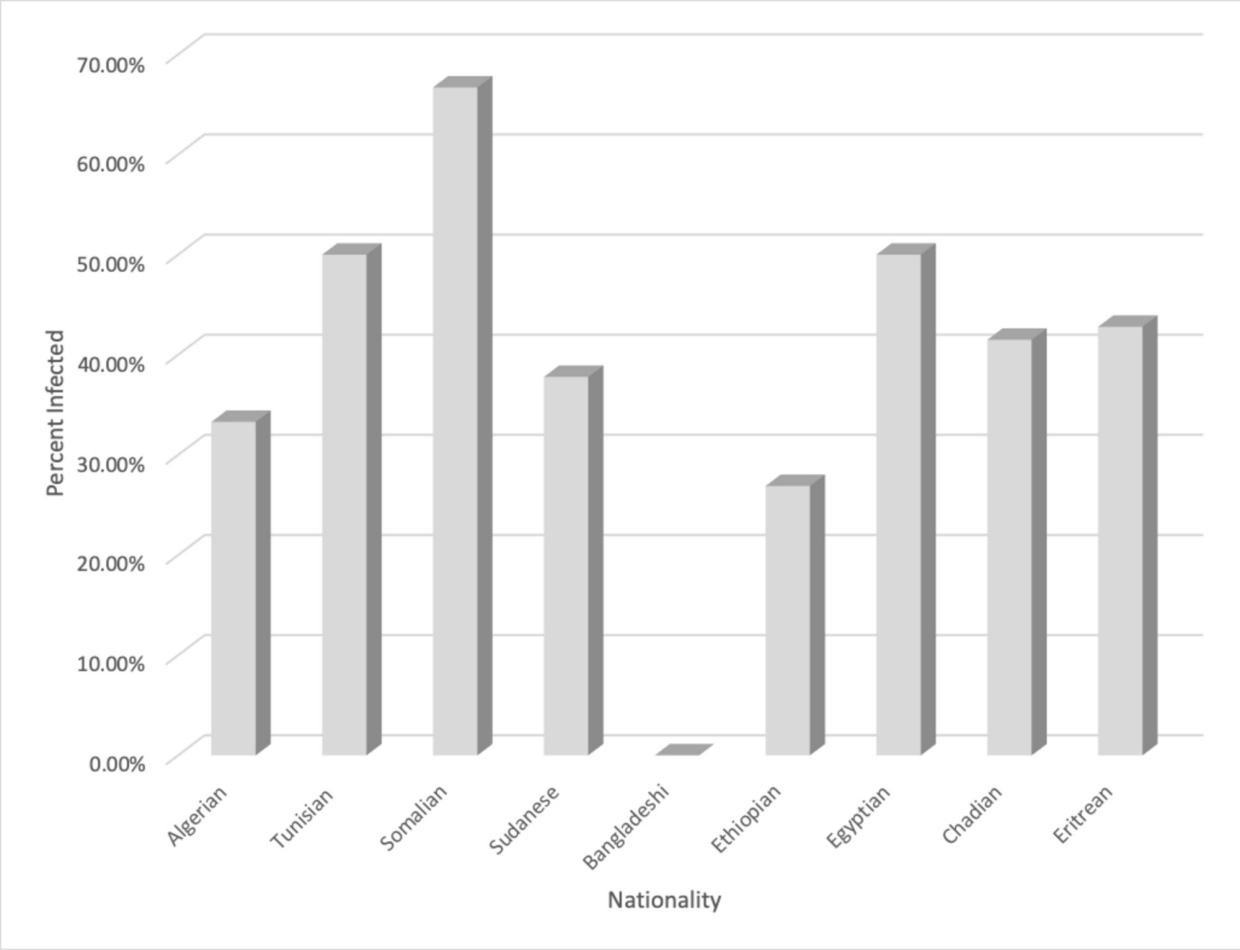
Comparative percentage prevalence of intestinal infestation amongst people belonging to different nationalities

Our study also included the assessment of infested people belonging to various regions. As depicted in Table 4 and Figure 4, the highest prevalence of intestinal parasites was recorded in people with Somalian nationality (66.7%), followed by Tunisian and Egyptian nationalities (50%) while fewer parasitic infestation presented among Bangladeshi nationalities (0.00%).

**Table 4:** Distribution of parasites according to nationalities P-value = 0.449

Nationality	Eritrean	Chadian	Egyptian	Ethiopian	Bangladeshi	Sudanese	Somali	Tunisian	Algerian	Total
Infested	6	17	4	14	0	42	6	4	2	95
Total number per Nationality	14	41	8	52	1	111	9	8	6	250

**Figure 5:**
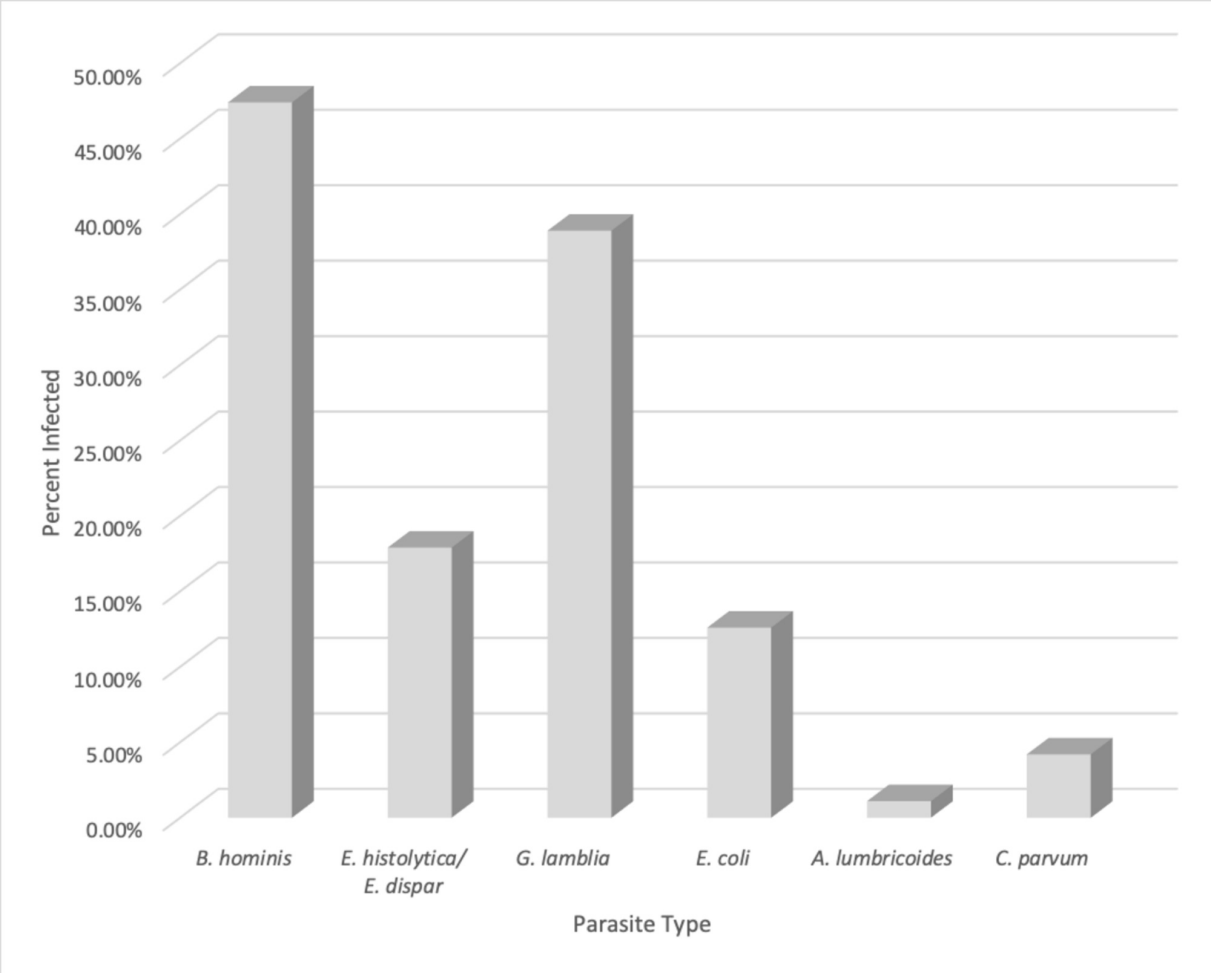
Comparative percentage assessment between various agents

There are no significant differences between nationality and parasitic transmissibility (P>0.449). It was also observed, as shown in Table 5 and Figure 5, that of all the tested samples, the most common intestinal protozoan parasite was Blastocystis hominis (B. hominis) with the highest prevalence at 47.4% (45), followed by Giardia lamblia (G. lamblia) at 38.9% (37), Entamoeba histolytica (E. histolytica) and Entamoeba dispar (E. dispar) at 17.9 %(17), Entamoeba coli (E.coli) at 12.6 % (12), and Cryptosporidium parvum (C. parvum) at 4.2% (4). Only the helminth parasite, Ascaris lumbricoides (A. lumbricoides), was detected at a low prevalence rate of 1.1% (1). There were significant differences in prevalence among B. hominis (P=0.00), G. lamblia (P=0.00), E. histolytica and E. dispar (P=0.00), C. parvum (P=0.036), E. coli (P=0.00), and no significant differences among A. lumbricoides (P=0.201), as seen in Table 5 and Figure 5.

**Table 5:** Prevalence of intestinal parasitic infestations amongst examined patients

Type of parasite	Infected (n=95)		p-value
	Number	(%)	
Blastocystis hominis	45	47.4	0.000
Entamoeba histolytica/ Entamoeba dispar	17	17.9	0.000
Giardia lamblia	37	38.9	0.000
Entamoeba coli	12	12.6	0.000
Ascaris lumbricoides	1	1.1	0.201
Cryptosporidium parvum	4	4.2	0.036

**Figure 6:**
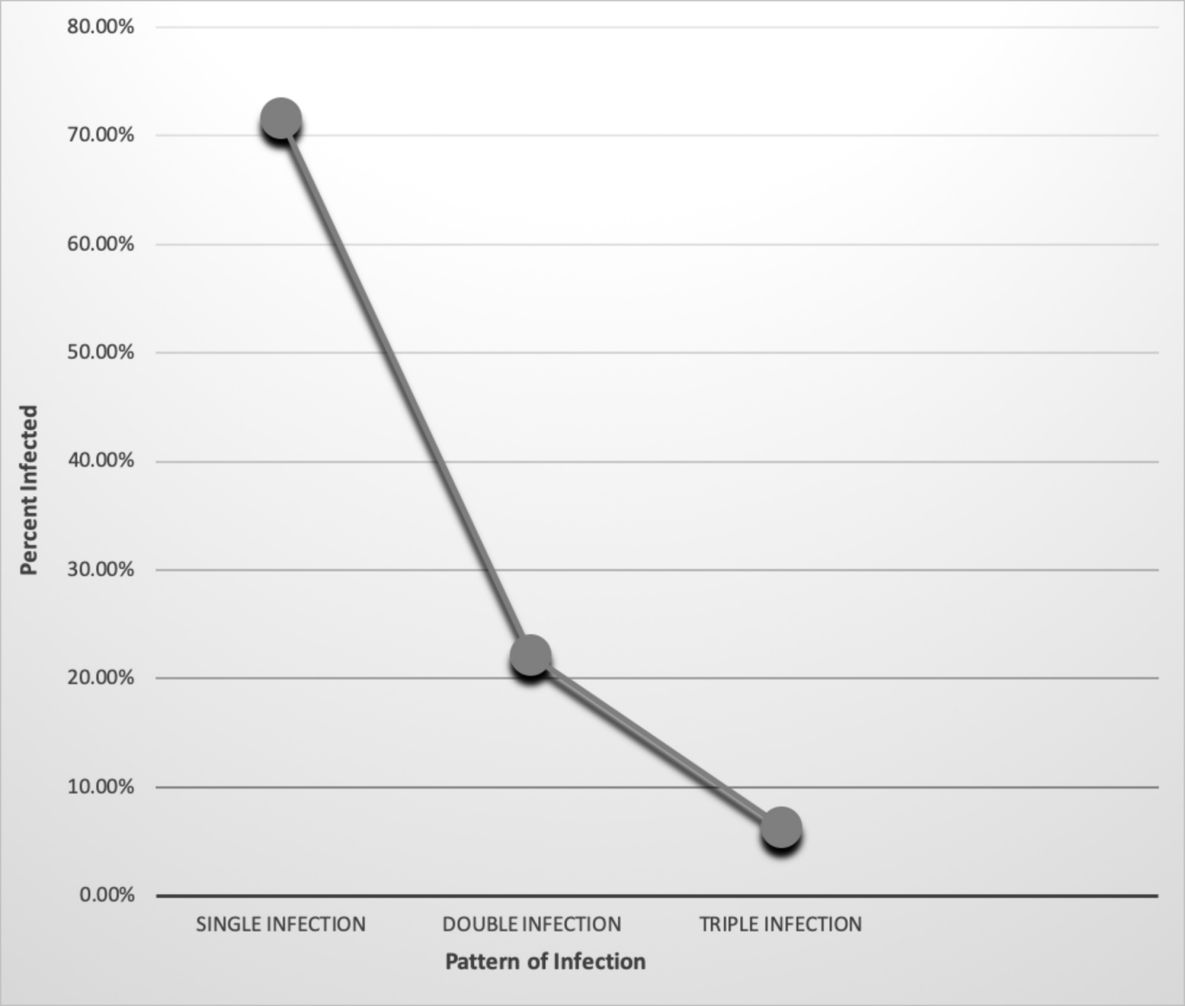
Percentage analysis of pattern of infection and prevalence

Our data, as shown in Table 6 and Figure 6, 71.6% showed infestation by a single parasite. Twenty-two point one percent (22.1%) of expatriate workers were found to be infested by two species of parasites and only 6% of the workers showed infestation by three species of parasites. The results showed that there was a high significant difference between prevalence and types of parasitic infestation (P=0.000). We also attempted to find out the relationship between the prevalence of parasitic infestation and diarrhea. 

**Table 6:** Pattern of intestinal parasitic infection among the studied sample

PATTERN OF INFECTION	INFECTED	
	NUMBER	%
Single infection	68	71.6
Double infection	21	22.1
Triple infection	6	6.3
Total	95	100

**Figure 7:**
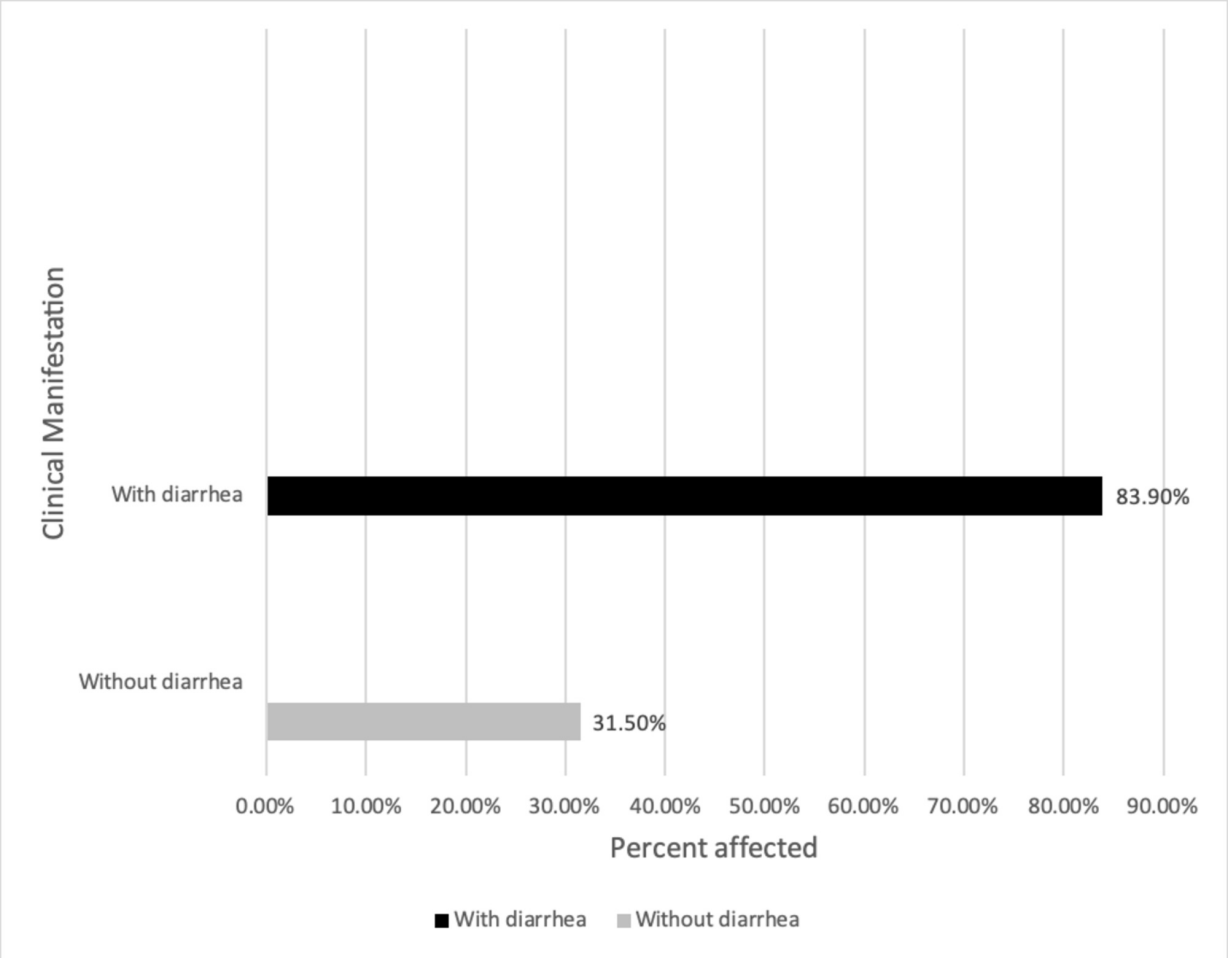
Percentage prevalence of intestinal parasitic infestation with and without diarrhea

It was observed that the prevalence of diarrhea with an infestation (83.9%) was greater than that of without diarrhea (31.5%), as seen in Table 7 and Figure 7. There was a highly significant difference, which was detected between the prevalence of parasitic infestation and diarrhea (P=0.000). Data were also analyzed based on the occupation.

**Table 7:** Relationship between the intestinal parasitic infection and diarrhea amongst various populations P-value = 0.000

Diarrhea	Total examined (n=250)	With parasitic infection (n=95)
With diarrhea	31	26 (83.9%)
Without diarrhea	219	69 (31.5%)
Total	250	95

**Figure 8:**
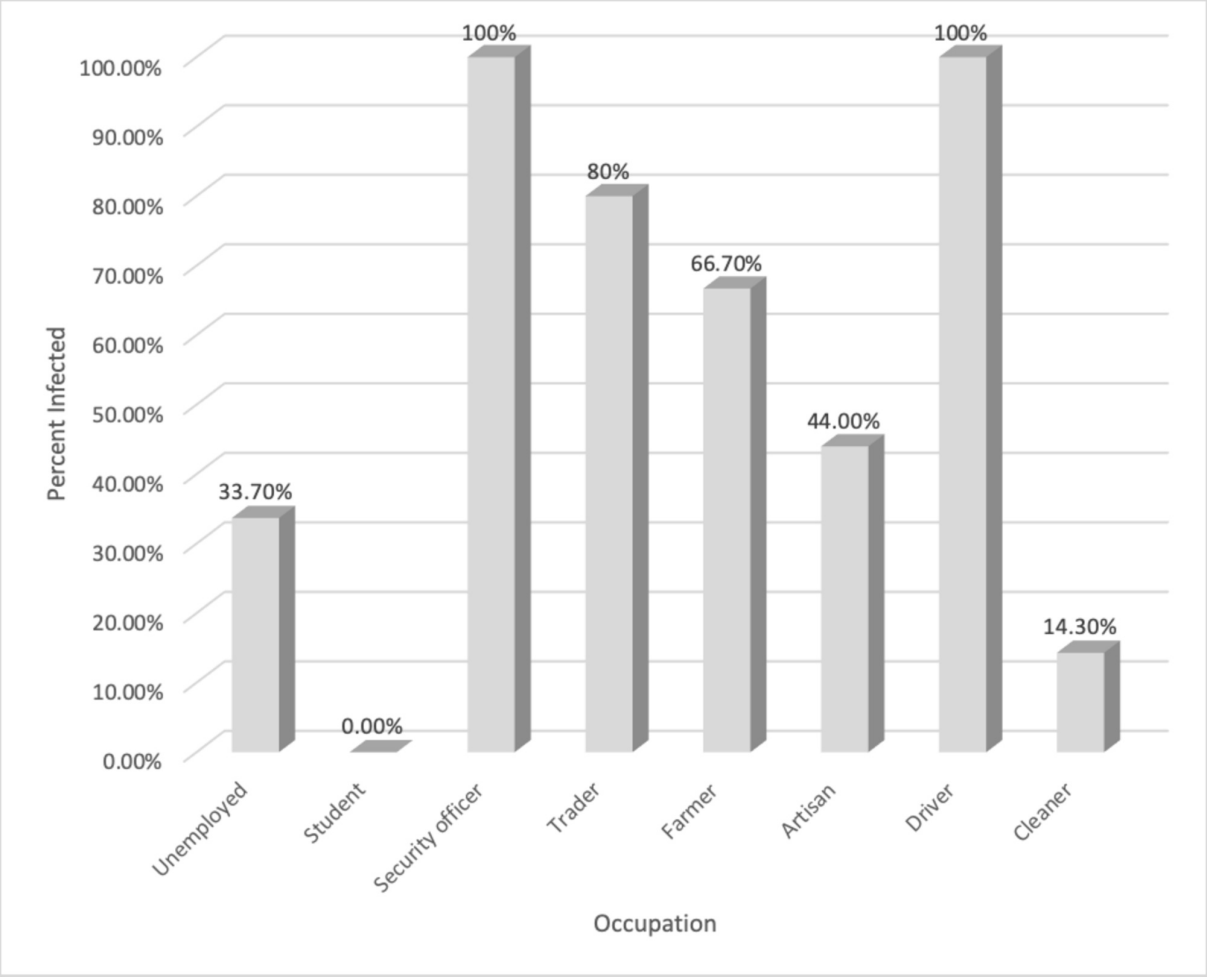
Percentile analysis of intestinal parasitic infestation within various occupations

As depicted in Figure 8 and Table 8, our results revealed that drivers and security officers showed a 100% prevalence of infestation while traders, farmers, artisan, unemployed, cleaners, and students showed 80%, 66.7%, 44%, 33.7%, 14.3%, and 0.00%, respectively. There was a significant difference between occupation and parasitic infestation (p>0.049).

**Table 8:** Relationship between the intestinal parasitic infestation and occupation amongst various populations P-value = 0.049

Occupation	Unemployed	Student	Security officer	Trader	Farmer	Artisan	Driver	Cleaner	Total
Infested	64	0	2	4	12	11	1	1	95
Total number for each occupation	190	2	2	5	18	25	1	7	250

Data from Table 9 show the length of parasitic infestation and employment length. The data suggest a higher percentage of parasite infestation when the length of employment was less than one year (41%) in comparison to when the parasitic infestation was greater than one year (36%). Furthermore, the non-parasitic infection had a greater risk in cases where employment length was more than one year.

**Table 9:** Relationship between the infestation of intestinal parasites and employment length P-value =0.425

Years of residence	Number examined	Parasitic infestation	Non-parasitic infection
Less than 1 year	100	41 (41%)	59 (59%)
More than 1 year	150	54 (36%)	96 (64.0%)
Total	250	95 (38.0%)	155 (62.0%)

Table 10 illustrates infected patients in combinations with other disorders. Of the 193 patients that were infested with parasites, 72 patients were non-infected with other disorders, two patients were infected with hepatitis C virus (HCV), seven with human immunodeficiency virus (HIV), two with atopic dermatitis, four with abdominal pain, and eight with hepatitis B surface antigen (HBsAg).

**Table 10:** Infected patients in combination with other disorders P-value =0.016

Risk Factors	Non-infected	HCV	HIV	Allergic cutaneous (atopic dermatitis)	Abdominal pain	HBsAg	Total
Parasitic Infestation	72 (37.3%)	2 (100%)	7 (87.5%)	2 (50.0%)	4 (19.0%)	8 (36.4%)	95 (38%)
Total Number per participant	193	2	8	4	21	22	250

## Discussion

Intestinal parasitic infestations and the associated diseases are still prominent in the tropical and sub-tropical areas of the world and are more common in developing nations. This high rate of infestation might be attributed to poor hygiene standards in developing countries. The prevalence of intestinal parasites is largely due to poor personal hygiene practices and environmental sanitation, inadequate supply of safe water, and ignorance of health-promoting practices. The prevalence of intestinal parasitic diseases is higher among expatriate workers due to poor sanitation. The current study showed that the prevalence of total intestinal parasites reached 38%. In one of the previous studies in the district of Derna, Libya, the prevalence of infestation was found to be 31% [11]. This prevalence rate was found to be higher as compared to the other studies in Zawia, Libya, with 10.6% [12], and 12.88% in Benghazi, Libya [13]. Another study conducted among school children by Ben Musa in 2007, reported a prevalence of 14.6% [14]. This prevalence is also higher than the study done in other countries, such as in Saudi Arabia, where the prevalence was 29.4% [15], Nepal, with a prevalence of 18.4% [16], and Brazil, with a prevalence of 22.3% [17].

Different studies conducted in Alhag Yousif Area, Khartoum, Sudan [18] and southern Sudan [19] showed a higher prevalence of 64.4% and 66%, respectively. The present study showed that intestinal protozoan parasitic infestations were more prevalent than helminthic infestations, in corroboration with the previous studies [20-23]. The present study revealed that the highest proportion of parasitic infestation was in age groups ranging above 45 years [21,23]. Furthermore, the present study found that there is no statistical significance (P=0.581) between age groups and the rate of infestation. Our data agrees with the findings of Okyay P et al., 2004 [24], Sadaga, 2007 [11], and Chandrashekhar et al., 2005 [25]. In this study, the prevalence of an intestinal parasitic infestation of expatriate workers in males was higher than in females. The percentage was 42.5% and 20 % in females and males, respectively [24-25].

## Conclusions

The prevalence of parasitic infestation was relatively high (38%) and was affected by individual hygiene. Therefore, comprehensive healthcare education aimed at reducing parasitic infestation is needed. Social awareness through media and other avenues provides an appropriate gateway for ensuring all individuals, including expatriate workers, are educated. It is recommended that all expatriate workers should be checked and treated, if necessary, upon arrival to Libya. The combination of adequate hygiene practices and preventative measures in expatriate workers could enable a decrease in the prevalence of parasitic infestations.
